# Research interest and activity among medical students in Gothenburg, Sweden, a cross-sectional study

**DOI:** 10.1186/s12909-016-0749-3

**Published:** 2016-08-26

**Authors:** Marit Stockfelt, Lars Karlsson, Caterina Finizia

**Affiliations:** 1 Institute of Medicine, Department of Rheumatology and Inflammation Research, Sahlgrenska Academy, University of Gothenburg, Guldhedsgatan 10A, 405 30 Gothenburg, Sweden; 2 Institute of Neuroscience and Physiology, Department of Clinical Neuroscience and Rehabilitation, Sahlgrenska Academy, University of Gothenburg, Gothenburg, Sweden; 3 Department of Otorhinolaryngology, Sahlgrenska Academy, University of Gothenburg, Gothenburg, Sweden

**Keywords:** Medical student, Research, Recruitment, Physician, Scientist

## Abstract

**Background:**

The proportion of physicians undertaking doctoral studies is decreasing. Early recruitment of medical students could counteract this trend. This follow-up survey investigated research interest and activity among medical students at the Sahlgrenska Academy, Gothenburg, Sweden.

**Methods:**

A questionnaire was administered to all medical students at the Sahlgrenska Academy, as a follow-up to a 2006 survey. The Mann-Whitney *U* test was used for ordinal variables and the Fisher exact test for categorical variables. Data from Statistics Sweden was used to analyse the number of PhDs awarded to individuals who earned a medical degree in 2000–2012.

**Results:**

Of the students, 16 % were already conducting and another 36 % wanted to conduct research during their studies. The interest was at the same level compared to 2006. The main reasons for conducting research consisted of an interest in scientific problems or the research topic, a wish for personal development or intellectual stimulation. Students engaged in research reported lack of time, increased workload and less time to study as hindering factors.

**Conclusions:**

Recruitment could be improved by offering improved and regular information, clarifying career paths, broadly announcing available projects, and creating new and expanding existing research programmes. The potential for recruitment of Gothenburg medical students to research is substantial, but students are hampered by lack of time, lack of supervisors and lack of information.

**Electronic supplementary material:**

The online version of this article (doi:10.1186/s12909-016-0749-3) contains supplementary material, which is available to authorized users.

## Background

The proportion of physicians undertaking doctoral studies has decreased progressively over the last years, both in Sweden [1] and internationally [2, 3]. Studies from the US report that the number of US medical students interested in research has decreased, as has the percentage of US medical doctors among those receiving National Institute of Health (NIH) government grants [4, 5]. The number of US physicians with research as their main professional activity is also decreasing [6]. In Sweden, the proportion of physicians among PhDs, and PhD students at Swedish medical faculties, has declined progressively since the beginning of the last decade. Moreover, the median age for Swedish physicians to finish their doctoral studies is currently 41 years, as compared with 34 years for other doctoral degrees [1].

Physicians trained in the scientific disciplines and the field of clinical medicine are essential for bringing patient-oriented research questions into focus, and bridging the gap between basic and clinical sciences [7, 8]. A shortage of physicians in research could in the future have negative consequences for academia, clinical research and health care [1], undermining the translation of basic research into patient care [8, 9]. To counteract this trend, early recruitment of medical students has been proposed, and medical students engaged in research during medical studies conduct more postgraduate research compared with their peers in the US [10–12] and the Netherlands [13]. Research conducted by medical students has been shown to be productive in terms of publications, as reported from Norway [14] and Germany [15].

The Swedish medical education spans 5.5 years, of which half a year at the end of the medical programme is dedicated to writing a research Master’s thesis mandatory for all students as part of the curriculum. An additional 1.5 years of internship is required to obtain a licence to practise medicine. With the PhD education in Sweden currently lasting 4 years, this adds up to a total of 11 years to become a licensed physician holding a PhD. One of six medical schools in Sweden is located in Gothenburg at the Sahlgrenska Academy. For extracurricular research, a part of the students are involved in the ‘Research assistant programme’ which was initiated in 2009 to stimulate research among medical students. Currently the programme offers ten positions per year [16]. Students are accepted for a 3-year period with scheduled research activities, such as lectures and presentations, and financial compensation for part-time research in parallel with their medical studies. Similar research stimulating projects can be found at other Swedish universities, in the forms of summer research programmes and research preparatory courses.

In light of this and other efforts made to stimulate research engagement among medical students in Gothenburg, we have conducted a follow-up survey to investigate the progression of research interest and extracurricular research activity among medical students.

## Methods

### Study design and participants

An anonymous questionnaire composed of both open and closed questions was administered electronically to all medical students at the Sahlgrenska Academy during the fall of 2012 and the spring of 2013 (Additional file 1). The questionnaire was partly based on a previous survey conducted in Gothenburg in 2006 that examined research interest and extracurricular research activity among medical students [17]. Research was defined as participating in a medical research project on scholarship, as a PhD student or as a researcher during and/or between semesters, but excluding the Master’s thesis at the end of the medical programme. Answers were required for all questions except for the open response questions. Parts of the data have previously been published in Swedish [18]. The research was performed in compliance with the Helsinki Declaration and approval from the Faculty was given.

Questions were graded on a 10 point scale, with one representing to a very low degree, and ten representing to a very high degree. To questions with alternatives, the respondents were asked to choose one or multiple alternatives they agreed with, as well as to add their own alternatives. For open response questions, answers were systematically analyzed and assigned into mutually exclusive categories based on the respondents’ answers. From the most common categories, quotes were chosen as examples.

### Statistics

A 10-point numerical scale was used for graded answers. When comparing categorical variables, the Mann-Whitney *U* test was used for ordinal variables and the Fisher exact test for categorical variables.

Data from Statistics Sweden (SCB), that were based on Swedish Register of Education data, were used to analyse the total number of awarded PhD degrees in medicine (orientation code 731 according to the Swedish Educational Terminology, SUN-2000) for persons holding a medical degree in Sweden for each calendar year from 2000 to 2012.

## Results

### The participants

The questionnaire was completed by 471 students, resulting in a response rate of 42 %. Of the respondents, 264 were female and 207 male. The distribution was comparable across the semesters. The proportion of women and men answering the questionnaire (56 and 44 % respectively) was consistent with the gender distribution in the medical programme in the autumn of 2012 (53 and 47 % respectively). The median age was 25 years, with 14 % of respondents being older than 30 years. Most of the students were living alone without children (54 %) or were married/cohabiting without children (35 %) and 10 % of respondents had children (Table 1).

**Table 1:** Gender, age and family situation of students conducting/not conducting active research projects

Gender	Median age (yrs)	Active research		Family situation			
		Yes *N* (%)	No *N* (%)	Single without children *N* (%)	Married/Cohabiting without children *N* (%)	Single with children *N* (%)	Married/Cohabiting with children *N* (%)
Female	24	38 (14 %)	226 (86 %)	139 (53 %)	106 (40 %)	1 (0 %)	18 (7 %)
Male	26	37 (18 %)	170 (82 %)	117 (57 %)	61 (29 %)	3 (1 %)	26 (13 %)
Total	25	75 (16 %)	396 (84 %)	256 (54 %)	167 (35 %)	4 (1 %)	44 (9 %)

### Are medical students interested in research?

The proportion of students with active research projects was 16 %. Another 36 % reported interest in conducting active research during their medical studies. The interest in scientific issues was substantial and did not vary between semesters. Respondents scored an average of 7.4 on a scale from 1 (very little interest) to 10 (very great interest). Male students indicated a slightly greater interest, scoring 7.7 compared with 7.1 for female students (*p* < 0.01).

**Fig. 1:**
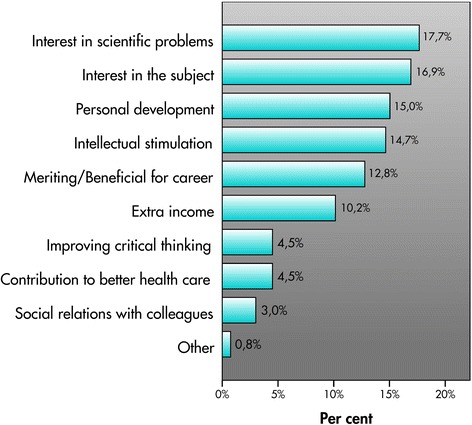
Reasons for medical students to conduct research

The major reasons for conducting active research were an interest in scientific problems, an interest in the research topic, and a desire for personal development or for intellectual stimulation (Fig. 1). Students who expressed interest but were not actively conducting research indicated similar reasons. Among them, a higher proportion wanted to contribute to better health care and acquire critical thinking skills, while fewer expressed a wish for an extra income.

**Fig. 2:**
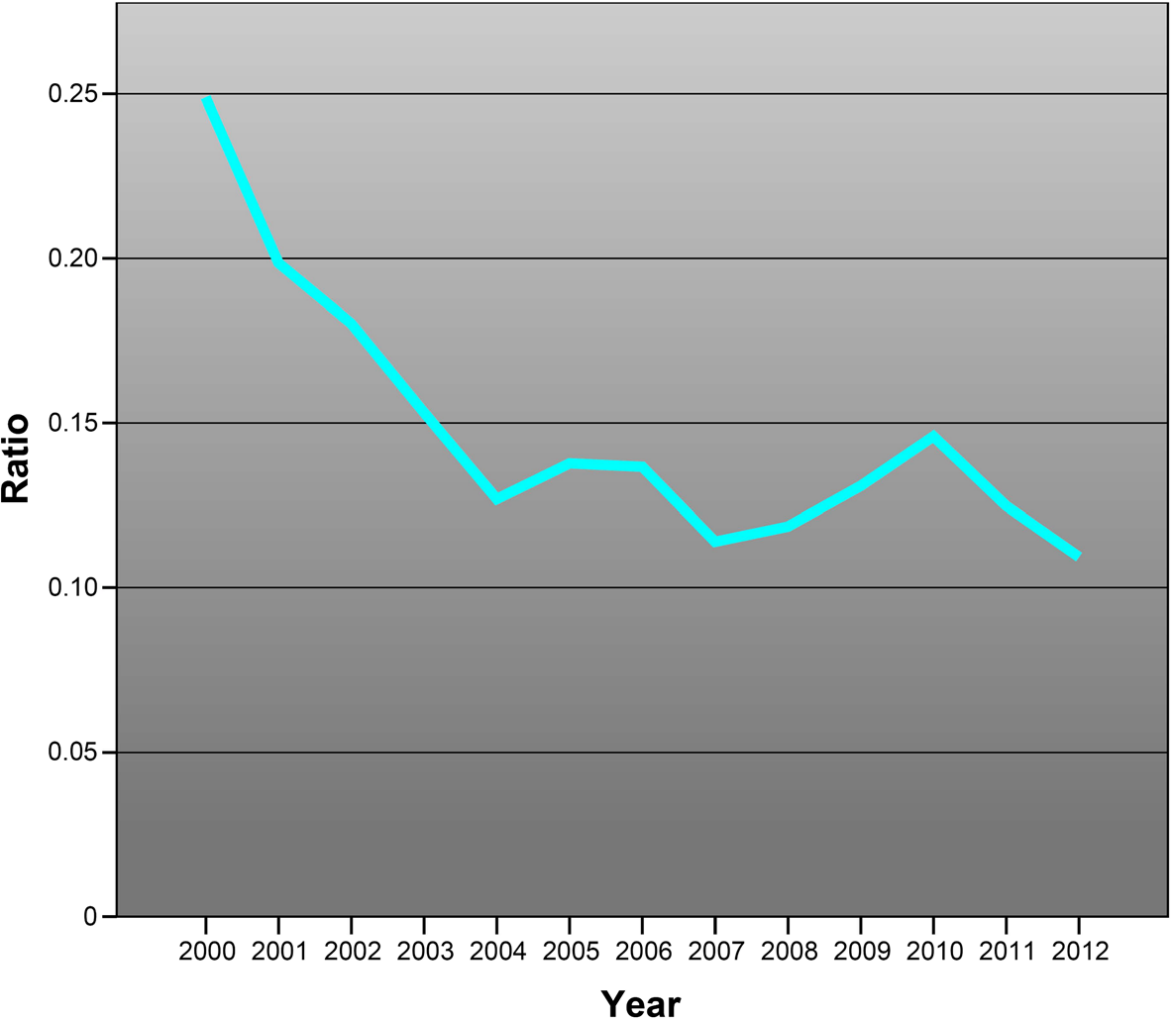
Ratio between PhD degrees awarded to persons holding a medical degree and number of medical licences issued per year in Sweden for the period 2000–2012

Statistics produced by SCB show that the number of physicians finishing their PhD per year was largely unchanged between the years 2000–2012. By contrast, statistics from the Swedish National Board of Health and Welfare show that the number of Swedish medical licences issued per year almost doubled during the same period, from 1185 to 2186 (Fig. 2).

### What are the disincentives for doing research?

Lack of time (23 %), increased workload (22 %) and less time to study (16 %) were disincentives reported by students engaged in research. Inadequate financial compensation was considered by a minority (9 %) to be a disadvantage. Other reported disincentives were delayed income rise, slower career development, and research not being as meriting as clinical work.

**Fig. 3:**
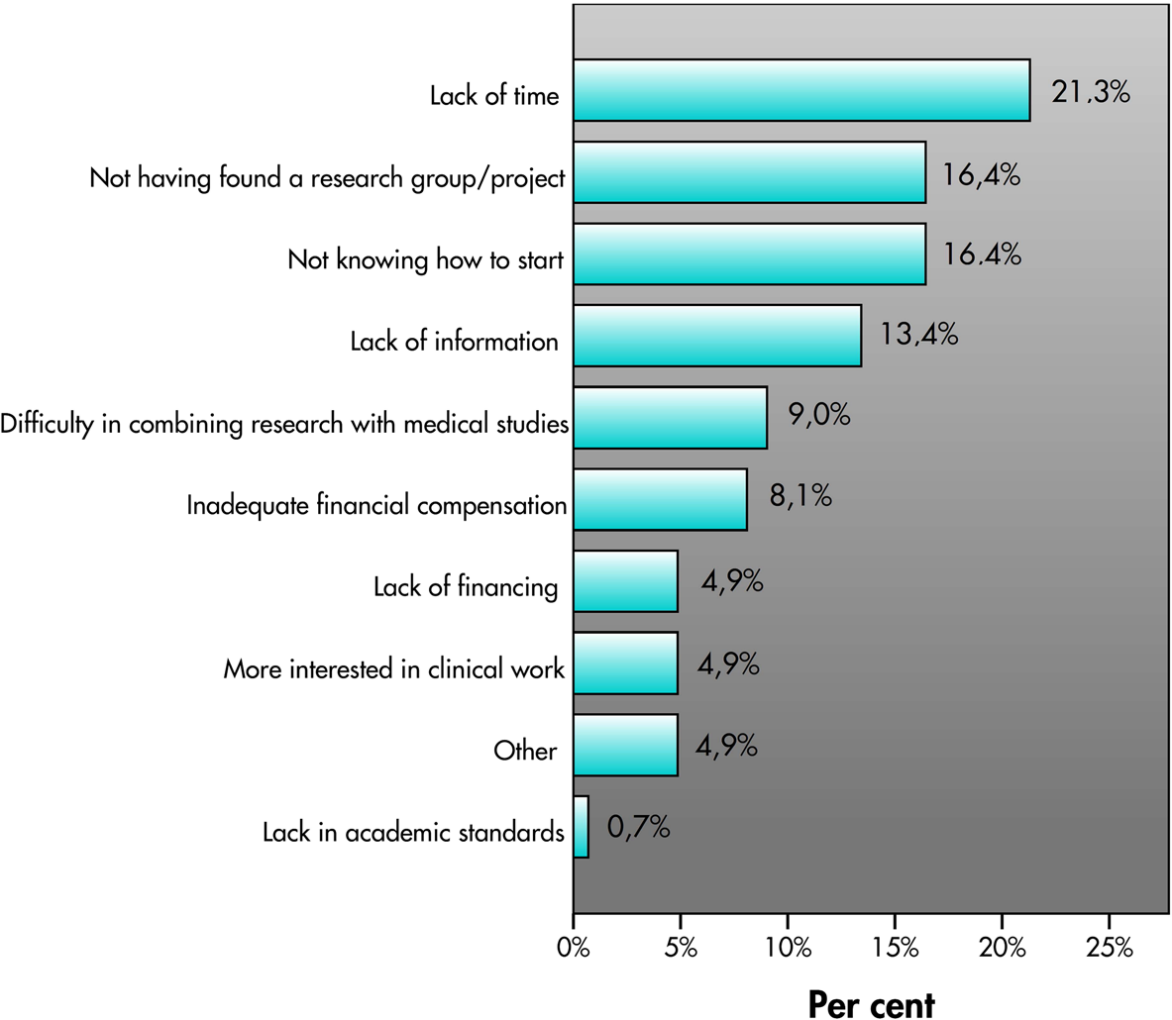
Hindering factors for medical students who wished to conduct research

Students who were interested in research but did not have an active research project mainly identified lack of time, followed by not knowing how to start and not having found a research group or supervisor, as hindrance to starting a research project. Many students also indicated lack of information, difficulty in combining research with medical studies (9 %) and inadequate financial compensation, and a small percentage also indicated lack of finance, ‘more interested in clinical work’ , and ‘other’ (Fig. 3).

The students who were not interested in conducting research often identified time constraints (40 %) as the reason for not conducting research, followed by lack of interest (15 %), economic aspects (9 %) and family situation (9 %). These students felt that improved information (37 %), better financial compensation (27 %) and better work conditions in the future (23 %) would encourage more medical students to engage in research.

### How do students get involved in research?

Most students actively engaged in research established contact by approaching the research team on their own. Some students continued after having conducted research on summer breaks, or after attended the Research assistant programme, while a smaller portion continued after completing the half year Master’s thesis for the medical degree, or were approached by the group or a teacher, or stated “other” way of being recruited than those suggested (Table 2). The lecturers’ commitment and personal contacts were important factors that got the students to do research, as were scholarships or having fellow students involved in research. Half of the students (49 %) reported that a lecturer had attempted to recruit them to research during lecture, while 11 % reported that this had occurred in private conversation. Only 14 % felt that they had received enough information and knowledge about conducting research during medical school. The Research assistant programme was known and had an impact on research interest for three quarters of the students. The positive influence was regardless of whether the students were conducting active research or not, with responses averaging at 5.7 and 4.3 respectively on a scale from 1 (very low influence) to 10 (very high). The proportion of students conducting active research increases during later semesters, with 8 % in the second semester and 19 % in the eleventh semester. Of the students who were not conducting active research, most reported willingness to engage in research in the future, and of those who were conducting active research, a majority of 80 % considered undertaking PhD studies in the future.

**Table 2:** Pathways for involvement in research during medical studies

	Count	Percentage
Approached the research group	45	40.2 %
Other	25	22.3 %
Continued after research activity on summer breaks	20	17.9 %
Participated in the Research assistant programme	14	12.5 %
Continued after completing thesis project for medical degree	5	4.5 %
Approached by research group or recruited by teacher	3	2.7 %
Total	69	100.0 %

### What kind of research do the students wish to pursue?

Basic science research projects, defined as being performed mainly in a laboratory environment, were most common (45 %), while a third of the students worked on clinical projects (32 %), defined as research projects that mainly involved human participants. The remaining respondents were involved in projects with both basic science and clinical aspects (23 %). Among the students who wished to do research actively, only one-tenth were interested in basic science research (9 %), while one-third preferred clinical research (30 %) and half favoured a project that included both basic science and clinical aspects (51 %).

### How do students combine research and medical studies?

In general, students engaged in research found it intermediately difficult to combine medical studies with active research, with an average response of 4.5 on a scale of 1 (very easy to combine) to 10 (very difficult). Common forms of compensation for extracurricular research activity were salary (47 %), scholarship (33 %) or no compensation at all (15 %). Students willing to engage in research were interested in conducting research to a high extent between semesters, with an average score of 7.3 on a scale of 1 (very little extent) to 10 (very high).

### How should we improve recruitment to research?

Students were asked in an open response question how recruitment could be improved. Students conducting research suggested that recruitment could be improved by offering financial compensation, informing and inspiring students, and clarifying career paths. Students interested in engaging in research requested information about research during lectures, and public announcements of research projects and groups. Other suggestions included expanded programmes and opportunities such as the Research assistant programme, and scholarships for summer research. Financial compensation, information and transparency in the recruitment process were also desired.

## Discussion

### Strong interest in research among medical students

The present study shows that a large percentage of medical students in Gothenburg are interested in conducting research. The interest in scientific issues in our study population was at the same level as reported in the previous questionnaire study in 2006 [17] with a remaining gender difference, where male students indicated a slightly higher interest compared with female students, as also seen elsewhere [19]. Our survey showed that the proportion of students conducting active research increases during later semesters, and of the students who were not conducting extracurricular research, most reported willingness to engage in research in the future.

### Lack of time, information, and research group are hindering factors

The main obstacles to engaging in research identified in this study are lack of time, lack of information, and lack of supervisors. However, lack of compensation, which has previously been proposed as a major obstacle [20], did not come up as one of the more important factors (Fig. 3). Possibly this becomes important once a research project is established, but by that time the majority of the students with active research projects in our study already had some form of financing. This study confirms the importance of continuously offering information about what research involves and how to begin a research career. Lack of time came up as an important barrier, and students not interested in conducting research often identified time constraints as the reason. Students at the Sahlgrenska Academy do not get any protected time for research. However, recently half a year has been dedicated for pursuing a Master’s thesis at the end of the medical education as a part of the curriculum. It might be interesting to follow whether this has an impact on extracurricular research activity among medical students.

### Difficulty in finding a research group

In our survey, a strong interest in research was reported, as 36 % of those respondents not already conducting research reported willingness to engage in research during medical studies. However, we identified a gap between interested students and accessible supervisors. As one student wrote in free text, ‘Give us the chance to start doing research. The whole class would probably come running.’ The potential for improvement here lies primarily in improved contact between research groups and interested students. A recurring suggestion from the students was to have a central website where available projects and research groups could be publicly announced for students to apply to, a feature that has been requested by students in a Hawaiian study as well, where a vast majority of students were positive to an online searchable database of research projects [21]. A first step in the mediation of research projects has been introduced at the Sahlgrenska Academy in the form of a project database for Master’s thesis. This tool could easily be adapted for the use of students looking for a long-term engagement in research.

### The Research assistant programme enhances research interest throughout the medical programme

This survey included 24 out of the 30 medical students who at the time were active in the Research assistant programme. This means that two-thirds of the students conducting research were doing research outside of the Research assistant programme. In our study, many of the students requested an expansion of the programme. It is also interesting to note that the Research assistant programme seems to have a positive influence on research interest both among students doing active research and among other students. A similar medical student research programme in Norway has been reported to have increased the recruitment of physicians to research [14]. The University of Queensland in Australia has implemented the Clinical Scientist Track, a research intensive programme for medical students to pursue a research Master’s degree alongside their medical degree, which has led to a majority continuing research as PhD students [22]. In Canada, apart from the recently cancelled joint MD/PhD program [23], there is since 1995 a Clinician Investigator Program through which clinicians engage in research training concurrently with their postgraduate medical education. This program has been recognized as an important mechanism for producing highly qualified clinicial investigators [24]. Furthermore, participation in a research training programme for medical students at the Howard Hughes Medical Institute, in Maryland in the US, has been shown to increase the likelihood of receiving NIH post-doctoral support [11]. Research experience during studies at three different medical schools in the US has also been strongly associated with post-graduate research involvement [12].

### Basic science and clinical research

An acute shortage of medical students at the basic science stage has previously been described [1, 25]. We found a decreased interest in basic science projects during the early semesters, which is consistent with a previous study from 2006 [17]. Almost half the students conducting active research were conducting research for a basic science project, whereas only one-tenth of the students wishing to do research were interested in basic science research. Most students were especially interested in a combination of basic science and clinical research. Clinical research seems to be difficult to initiate during the early semesters, with the proportion of students doing clinical research projects increasing with time (from 0 % in the second semester to 11 % in the eleventh semester). The reverse is true for the proportion of students in this study working on basic science projects (8 % in the second semester and 2 % in the eleventh semester). Several students commented that an enhanced opportunity for clinical research during the early semesters would increase their interest to start doing research. The Swedish Research Council reported in 2003 that the research interest among medical students increases during later semesters. However, in our study, the interest in engaging in research did not seem to be dependent on the semester.

### Importance of early recruitment

A previous study of the Research assistant programme shows that medical students who begin research in pre-clinical semesters express stronger long-term research interest compared with students who start later [25], and the same pattern has been seen in early recruitment of medical students to surgery [26]. In light of substantial retirement of senior researcher physicians and a high median age for medical doctors gaining their PhD degree, efforts should be made for early recruitment of students to research. Lack of information about research and lack of availability of projects and research groups risks delaying recruitment to clinical research. This study reveals an opportunity for targeted and expanded efforts during the early semesters of medical school to improve information on current basic science and clinical research.

### High demand for recruitment

Increased recruitment of young physicians involved in research is required. Of the more than 6000 physicians in Sweden with a PhD, only 9 % are under 40 years [1]. We are facing retirements of senior research physicians, and the annual number of new PhD physicians is not sufficient for re-growth of qualified supervisors and clinical researchers. In a German study, medical student research activity has been shown to significantly increase output of publications at the faculty level [15].

Suggestions from our respondents as to how recruitment can be increased were to stimulate interest in research, guide interested students into active research, and support students who are already engaged in research. Students who were not interested in research indicated that lack of time is a hindering factor, and one way to address this would be to increase acceptance in the medical programme of participation in research group meetings and laboratory work during the studies. Students doing active research likewise considered lack of time as a disadvantage, with the extracurricular research activity resulting in increased workload and decreased study time. Furthermore, the financial compensation for research participation may be seen as inadequate, and scholarships for summer research and the Research assistant programme may be important incentives. It should be noted that although financial compensation did not feature as a crucial hindering factor, more structured programmes such as the Research assistant programme were requested and such programmes could constitute important incentives.

### Substantial recruitment potential

This questionnaire was sent to all students enrolled at the Sahlgrenska Academy. The response rate was low, with 42 % of students responding. Self-selection bias is possible, with students more interested in research being more likely to answer a questionnaire about the subject, meaning that the actual interest and engagement in this population may be overestimated. However, from the answers we can conclude that there is a substantial recruitment potential among Gothenburg medical students, with a large number of medical students across all semesters interested in conducting active research during their medical studies. The students are hindered mainly by a lack of information about what research entails and how to get in contact with research groups, and not so much by lack of financial compensation. Through targeted initiatives to stimulate research engagement, such as improved information, we may have an opportunity early on to recruit a large number of medical students to research and thus secure the need for physicians in research in the future.

## Conclusion

The proportion of physicians undertaking doctoral studies has decreased progressively over the last years, both in Sweden and internationally. To counteract this trend, it is important to investigate and improve recruitment of medical students to research. This study was designed to investigate research interest and engagement of medical students in Sweden in 2012, as a follow-up to a similar study done in 2006.

For increased and early recruitment of medical students, efforts could be directed towards offering improved and regular information about conducting research, publicly announcing available research projects, and creating and expanding research programmes for motivated medical students. Along with improved conditions and financial resources, these measures could help to accommodate the growing need for recruitment of medical students and physicians to research.

## Additional files

Additional file 1: English version of the questionnaire used in the study. (DOCX 109 kb) Additional file 2: Raw data from the questionnaire. (XLSX 214 kb) Additional file 3: Data from Statistics Sweden (SCB). (PDF 6 kb)
