# Supplementary material for: Research interest and activity among medical students in Gothenburg, Sweden, a cross-sectional study
Source: BMC Med Educ. 2016 Aug 26;16(1):226. doi: 10.1186/s12909-016-0749-3 (PMC5002212; doi:10.1186/s12909-016-0749-3)
Supplement: Additional file 1: — English version of the questionnaire used in the study. (DOCX 109 kb) [file 12909_2016_749_MOESM1_ESM.docx]

| 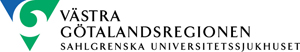 |  |
| --- | --- |

**Welcome to this survey about research interest**

**If you would like to comment on the questionnaire, or there are questions where suitable alternatives are lacking, it will be possible to write this at the end.**

**1. Which semester are you currently in?**

Semester 1-11

**2. Gender**

Female/Male

**3. Age _______**

**4. Family situation**

| 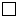 | Living alone |
| --- | --- |
| 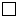 | Married/cohabitating |
| 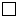 | Living alone with children |
| 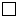 | Married/cohabitating with children |

**5. What is your interest for scientific questions?**

|  | 1 | 2 | 3 | 4 | 5 | 6 | 7 | 8 | 9 | 10 |  |
| --- | --- | --- | --- | --- | --- | --- | --- | --- | --- | --- | --- |
| Very small | 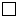 | 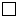 | 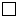 | 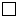 | 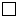 | 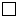 | 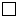 | 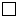 | 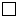 | 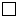 | Very large |

**6. Do you participate in a medical research project (apart from the Masters project as part of medical school) during and/or between semesters as a research student/researcher/as part of a scholarship.**

| 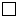 | Yes |
| --- | --- |
| 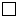 | No |

**Active researchers**

**(Answered by those answering Yes to question 6)**

**7. Is your project a clinical or basic science project?**

| 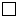 | Basic science |
| --- | --- |
| 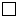 | Clinical |
| 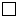 | Both basic science and clinical |
| 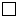 | Other ___________________ |

**8a. At which institute is your research based?**

| 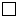 | Institute of Biomedicine |
| --- | --- |
| 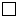 | Institute of Clinical Sciences |
| 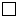 | Institute of Medicine |
| 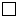 | Institute of Neuroscience and Physiology |
| 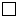 | Institute of Odontology |
| 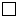 | Institute of Health and Care Sciences |

**8b. At what department in the Institute of Biomedicine is your research based?**

| 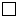 | Microbiology and Immunology |
| --- | --- |
| 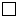 | Infectious Diseases |
| 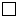 | Medical Biochemistry and Cell Biology |
| 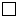 | Clinical Chemistry and Transfusion Medicine |
| 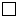 | Pathology |
| 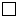 | Medical and clinical genetics |

**8b. At what department in the Institute of Clinical Sciences is your research based?**

| 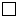 | [Anesthesiology & Intensive care](http://aic.gu.se/english) |
| --- | --- |
| 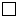 | [Biomaterials](http://biomaterials.gu.se/english) |
| 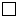 | [Dermatology and Venereology](http://dermven.gu.se/english) |
| 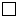 | [Gastrosurgical Research and Education](http://www.gastro.gu.se/Department%20of%20Gastrosurgical%20Research%20and%20Education/) |
| 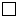 | [Surgery](http://surgery.gu.se/Department+of+Surgery) |
| 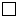 | [Obstetrics and Gynecology](http://www.obgyn.gu.se/english) |
| 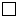 | [Oncology](http://oncology.gu.se/Department+of+Oncology) |
| 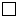 | [Orthopaedics](http://orthopaedics.gu.se/Department+of+Orthopaedics) |
| 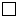 | [Pediatrics](http://pediatrics.gu.se/eDepartment+of+Pediatrics) |
| 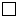 | [Plastic Surgery](http://plasticsurgery.gu.se/english) |
| 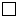 | [Radiation Physics](http://radfys.gu.se/eng) |
| 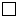 | [Radiology](http://radiology.gu.se/Department+of+Radiology) |
| 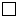 | [Urology](http://urology.gu.se/english) |
| 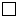 | [Otorhinolaryngology](http://www.clinsci.gu.se/Institute_of_clinical_sciences/otorhinolaryngology/?languageId=100001&contentId=-1&disableRedirect=true&returnUrl=http%3A%2F%2Fwww.clinsci.gu.se%2Foron-nasa-hals%2F) |

**8c. At what department in the Institute of Medicine is your research based?**

| 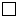 | Internal Medicine |
| --- | --- |
| 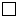 | Clinical Nutrition |
| 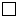 | Molecular and Clinical Medicine |
| 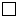 | Rheumatology and Inflammation Research |
| 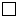 | Public Health and Community Medicine |
| 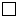 | Clinical Research, Innovation and Entrepreneurship |

**8d. At what department in the Institute of Neuroscience and Physiology is your research based?**

| 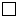 | Physiology |
| --- | --- |
| 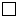 | Pharmacology |
| 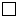 | Clinical Neuroscience and Rehabilitation |
| 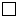 | Psychiatry and Neurochemistry |

**8e. At what department in the Institute of Odontology is your research based?**


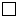
 Section 1 ([Endodontics](http://www.odontologi.gu.se/cat_dep_results.php?dep_list=4%205%2010), [Department of behavioral and community dentistry](http://www.odontologi.gu.se/cat_dep_results.php?dep_list=46), [Oral medicine and pathology](http://www.odontologi.gu.se/cat_dep_results.php?dep_list=16), [Oral & maxillofacial surgery](http://www.odontologi.gu.se/kirurgi), [Oral & maxillofacial radiology](http://www.odontologi.gu.se/radiologi), [Orofacial pain](http://www.odontologi.gu.se/cat_dep_results.php?dep_list=7))


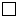
 Section 2 ([Oral biochemistry](http://www.odontologi.gu.se/cat_dep_results.php?dep_list=9), [Prosthodontics/dental materials science](http://www.odontologi.gu.se/cat_dep_results.php?dep_list=17), [Periodontology](http://www.odontologi.gu.se/cat_dep_results.php?dep_list=25%2026), [Dental laboratory technology](http://www.odontologi.gu.se/cat_dep_results.php?dep_list=18))

| 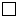Section 3 ([Cariology](http://www.odontologi.gu.se/cat_dep_results.php?dep_list=3), [Oral microbiology and immunology](http://www.odontologi.gu.se/cat_dep_results.php?dep_list=15), [Orthodontics](http://www.odontologi.gu.se/cat_dep_results.php?dep_list=22), [Pedodontics](http://www.odontologi.gu.se/cat_dep_results.php?dep_list=27%2028), Dental hygienist programme) |
| --- |

**8f. At what group or centre in the Institute of Health and Care Sciences is your research based?**

GPCC - University of Gothenburg Centre for Person-centred Care


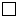


###
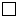
 Centre for Culture and HealthAcute and Critical Care Team (ACCT)

###
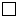
 Care Enviroment

###
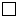
 Childbirth


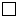
 Health Care Transition Research Group

###
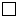
 Learning and leadership


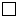
 Person-centered care in long-term condition


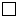
 The Palliative Care Research Group

**9. Do you at present take part in the Research Assistants Programme?**

| 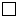 | Yes |
| --- | --- |
| 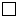 | No |
| 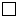 | I am not aware of the Research Assistants Programme |

**10. How did you begin your research?**

| 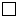 | I actively contacted the research group (kindly write below what made you do this) |
| --- | --- |
| 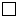 | I was contacted by the research group or recruited by a teacher |
| 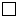 | I took part in a summer research project and continued thereafter |
| 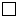 | I continued after the Masters project during Medical school |
| 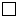 | I took part in the Research Assistants Programme |
| 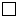 | Other_______________ |

**11. On average, how many hours each week do you spend on research during terms?**

**12. Are you co-author on an original paper in a peer reviewed journal?**

| 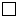 | Yes |
| --- | --- |
| 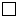 | No |

**13. Why do you do research?**

| 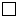 | Interest in scientific problems |
| --- | --- |
| 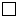 | Interest in the subject |
| 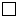 | Personal development |
| 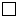 | Extra income |
| 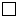 | Contribution to better health care |
| 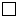 | Improving critical thinking |
| 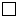 | Meriting/Beneficial for career |
| 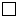 | Intellectual stimulation |
| 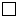 | Social relations with colleagues |
| 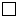 | Other_________________________________ |

**14. What is the level of difficulty in combining research and medical studies?**

|  | 1 | 2 | 3 | 4 | 5 | 6 | 7 | 8 | 9 | 10 |  |  |
| --- | --- | --- | --- | --- | --- | --- | --- | --- | --- | --- | --- | --- |
| Very difficult to combine | 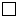 | 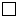 | 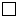 | 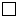 | 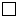 | 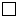 | 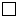 | 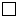 | 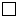 | 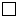 | Very easy to combine |  |
| Comments____________________________________ | | | | | | | | | | | | |

**15. What sort of compensation do you receive for your working hours?**

| 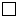 | Wages |
| --- | --- |
|  | Research assistant pay |
|  | Scholarship |
|  | No compensation |
|  | Other__________________________ |

**16. What advantages do you see for yourself in doing research?**

**17. What disadvantages do you see for yourself in doing research?**

**18. How are you as a researching student being met during medical school?**

|  | 1 | 2 | 3 | 4 | 5 | 6 | 7 | 8 | 9 | 10 |  |  |
| --- | --- | --- | --- | --- | --- | --- | --- | --- | --- | --- | --- | --- |
| Mainly negatively |  |  |  |  |  |  |  |  |  |  | Mainly positively |  |
| Comments____________________________________ | | | | | | | | | | | | |

**19. How do you think it would be possible to increase research interest in medical students who do not do active research at present?**

**20. Would you consider pursuing a PhD degree?**

|  | Yes |
| --- | --- |
|  | No |
|  | I am registered as a PhD student |
|  | I have attained a PhD degree |

**21. Are you interested in a research internship?**

|  | Yes |
| --- | --- |
|  | No |
|  | Do not know what that means |
|  | Undecided |

**Not doing research**

**(Questions given to those answering No to question 6)**

**22. How do you think researching students are being met by teachers during medical school?**

|  | 1 | 2 | 3 | 4 | 5 | 6 | 7 | 8 | 9 | 10 |  |  |  |  |
| --- | --- | --- | --- | --- | --- | --- | --- | --- | --- | --- | --- | --- | --- | --- |
| Mainly negative |  |  |  |  |  |  |  |  |  |  | Mainly positively | Undecided |  |  |
| Comments_________________________ | | | | | | | | | | | | | | |

**23. How could recruitment of medical students in to research be improved?**

**24. Would you like to do active research in the future?**

|  | Yes, before or during internship |
| --- | --- |
|  | Yes, after internship or during residency |
|  | Yes, after residency |
|  | No |

**25. Would you like to do active research during Medical school?**

|  | Yes |
| --- | --- |
|  | No |

**Would like to do research**

**(Questions given to those answering No to question 6 and Yes to question 25)**

**26. In what?
Explanation: With basic research we mean research in a mainly laborative environment with no large parts of patient studies. With clinical research we mean mainly patient studies with no large parts of laborative work.**

|  | Basic science |  | Clinical research |
| --- | --- | --- | --- |
|  | Basic science and clinical research |  | Do not know |
| Other_________________________ | | | |

**27. How many hours each week would you want to spend on research during semesters? _______________________**

**28. To what extent could you imagine doing research during study leaves (e.g. summer, study leaves etc)?**

|  | 1 | 2 | 3 | 4 | 5 | 6 | 7 | 8 | 9 | 10 |  |
| --- | --- | --- | --- | --- | --- | --- | --- | --- | --- | --- | --- |
| Small extent |  |  |  |  |  |  |  |  |  |  | Large extent |

**29. Why do you not do active research at this time?**

|  | Have not received enough information |
| --- | --- |
|  | Lack of time |
|  | Insufficient economic compensation |
|  | More interested in the clinic |
|  | Shortcomings in the academic environment |
|  | Difficulty combining studying with research |
|  | See no future in research |
|  | Do not know how to start |
|  | Lack of group or supervisor |
|  | Lack of funding |
|  | Other_____________________________________- |

**30. Why do you want to do research?**

|  | Interest in scientific problems |
| --- | --- |
|  | Interest in the subject |
|  | Personal development |
|  | Extra income |
|  | Contribution to better health care |
|  | Improving critical thinking |
|  | Meriting/Beneficial for career |
|  | Intellectual stimulation |
|  | Social relations with colleagues |
|  | Other______________________________ |

**31. Do you consider pursuing a PhD degree?**

|  | Yes |
| --- | --- |
|  | No |
|  | I am registered as a PhD student |
|  | I have attained a PhD degree |

**32. How do you think recruitment of medical students in to research could be improved? __________________________________**

**33. At which institute would you like to do research**

|  | \|  \| Institute of Biomedicine \| \| --- \| --- \| \|  \| Institute of Clinical Sciences \| \|  \| Institute of Medicine \| \|  \| Institute of Neuroscience and Physiology \| \|  \| Institute of Odontology \| \|  \| Institute of Health and Care Sciences \| |
| --- | --- | --- | --- | --- | --- | --- | --- | --- | --- | --- | --- | --- | --- |

**Do not want to do research**

(Questions given to those answering No to question 6 and No to question 25)

**34. Why do you not want to do active research?**

|  | Am not interested in research |
| --- | --- |
|  | Do not see any advantages for the career as a doctor |
|  | Lack of time |
|  | Family situation |
|  | Shortcomings in academic environment |
|  | Have not received enough information about research and what it entails |
|  | Economic aspects |
|  | Other____________________________ |

**35. What could increase your interest for doing research?**

|  | Better compensation |
| --- | --- |
|  | Better terms in the future |
|  | Better information |
|  | Other___________________________________ |

**Everyone**

(Questions given to everyone)

**36. Are research aspects covered in the course curriculum?**

|  | 1 | 2 | 3 | 4 | 5 | 6 | 7 | 8 | 9 | 10 |  |  |  |
| --- | --- | --- | --- | --- | --- | --- | --- | --- | --- | --- | --- | --- | --- |
| Very seldom |  |  |  |  |  |  |  |  |  |  | Very often | Do not know |  |

**37. Would you like to receive more research-based education during Medical school (that is, subjects touching on what research is, how it is done and how it affects medicine and society).**

|  | Yes |
| --- | --- |
|  | No |
|  | Don’t know |

**38. Have any teacher tried to recruit you in to research?**

|  | Yes, during a lecture |
| --- | --- |
|  | Yes, in personal communication |
|  | No |
|  | Other______________________________ |

**39. Have you during medical school or on your own received enough information and knowledge about what a PhD education entails?**

|  | Yes |
| --- | --- |
|  | Partly |
|  | No |

**40. To what extent has the possibility to participate in the Research Assistants Programme increased your engagement in research?**

|  | 1 | 2 | 3 | 4 | 5 | 6 | 7 | 8 | 9 | 10 |  |  |  |
| --- | --- | --- | --- | --- | --- | --- | --- | --- | --- | --- | --- | --- | --- |
| Small extent |  |  |  |  |  |  |  |  |  |  | Large extent | I am not aware of the Research Assistants Programme |  |

**41. What advantages do you see with doing research during medical school?**

|  | Critical thinking |
| --- | --- |
|  | Contacts |
|  | Facilitate medical studies |
|  | Extra income |
|  | Other______________________________- |

**42. What disadvantages do you see with doing research during medical school?**

|  | Time consuming |
| --- | --- |
|  | Insufficient economic compensation |
|  | Increased work load |
|  | Decreased time for studies |
|  | Other_____________________________________ |

**43. Other general comments or comments on the questionnaires. Did you e.g. miss any questions that you think should have been part of the survey? In that case, write the question/s and answer them here.**

_________________________________**Remember to press OK down to the right to register your answers!**

Thank you for your participation.

In case of questions, please contact

Lars Karlsson, Intern/PhD student, E-post:[lars.karlsson@neuro.gu.se](mailto:lars.karlsson@neuro.gu.se)

Marit Hansson, Intern/PhD student, E-post:[marit.hansson@gu.se](mailto:marit.hansson@gu.se)

Thank you!

Best regards, Lars and Marit
