# Supplementary material for: Research interest and activity among medical students in Gothenburg, Sweden, a cross-sectional study
Source: BMC Med Educ. 2016 Aug 26;16(1):226. doi: 10.1186/s12909-016-0749-3 (PMC5002212; doi:10.1186/s12909-016-0749-3)
Supplement: Additional file 3: — Data from Statistics Sweden (SCB). (PDF 6 kb) [file 12909_2016_749_MOESM3_ESM.pdf]

Number of Swedish PhD degrees per year in medicine* 2000-2012

Calendar year	Number of awarded PhDs	Number awarded PhDs holding an MD
2000	664	295
2001	656	264
2002	687	265
2003	721	260
2004	744	237
2005	780	249
2006	811	266
2007	749	252
2008	760	240
2009	826	256
2010	821	290
2011	758	265
2012	687	239

Source: Statistics Sweden (SCB); Educational Register (UREG).
Contact: Michael Karlsson (michael.karlsson@scb.se; tel.: +4619-17 64 81).

* Category #721 according to the SUN 2000-nomenclature

Number of Swedish medical licenses issued per year 2000-2012

Calendar year	Number of medical licenses issued	Ratio of PhD/MD per issued medical licenses
2000	1185	0,25
2001	1329	0,20
2002	1470	0,18
2003	1697	0,15
2004	1868	0,13
2005	1808	0,14
2006	1946	0,14
2007	2211	0,11
2008	2024	0,12
2009	1956	0,13
2010	1987	0,15
2011	2123	0,12
2012	2186	0,11

Source: National Board of Health and Welfare (Socialstyrelsen).
Contact: Birgitta Ollars (birgitta.ollars@socialstyrelsen.se; tel.: +4675-247 38 29).
